# SENS+: A Co-Existing Fabrication System for a Smart DFA Environment Based on Energy Fusion Information

**DOI:** 10.3390/s23062890

**Published:** 2023-03-07

**Authors:** Teng-Wen Chang, Hsin-Yi Huang, Cheng-Chun Hong, Sambit Datta, Walaiporn Nakapan

**Affiliations:** 1 College of Design, National Yunlin University of Science and Technology, Douliou 640, Yunlin, Taiwan; 2 School of Electrical Engineering, Computing and Mathematical Sciences, Curtin University, Bentley, WA 6102, Australia; 3 Parabolab, Bangkok 11000, Thailand

**Keywords:** internet of things, prototyping process, energy-saving, interactive design, user behaviors, ambient agents

## Abstract

In factories, energy conservation is a crucial issue. The co-fabrication space is a modern-day equivalent of a new factory type, and it makes use of Internet of Things (IoT) devices, such as sensors, software, and online connectivity, to keep track of various building features, analyze data, and produce reports on usage patterns and trends that can be used to improve building operations and the environment. The co-fabrication user requires dynamic and flexible space, which is different from the conventional user’s usage. Because the user composition in a co-fabrication space is dynamic and unstable, we cannot use the conventional approach to assess their usage and rentals. Prototyping necessitates a specifically designed energy-saving strategy. The research uses a “seeing–moving–seeing” design thinking framework, which enables designers to more easily convey their ideas to others through direct observation of the outcomes of their intuitive designs and the representation of their works through design media. The three components of human behavior, physical manufacture, and digital interaction are primarily the focus of this work. The computing system that connects the physical machine is created through communication between the designer and the digital interface, giving the designer control over the physical machine. It is an interactive fabrication process formed by behavior. The Sensible Energy System+ is an interactive fabrication process of virtual and real coexistence created by combining the already-existing technology, the prototype fabrication machine, and SENS. This process analyzes each step of the fabrication process and energy, fits it into the computing system mode to control the prototype fabrication machine, and reduces the problem between virtual and physical fabrication and energy consumption.

## 1. Introduction

### 1.1. Background

With the introduction of technology and software-based services, much research has been done to create smart environments that allow for seamless interaction between users and their immediate surroundings [1]. The focus of smart home/environment architecture is to develop practical collaboration between users and devices to optimize use and improve the quality of experiences and services [2]. The primary goal of a smart environment is to enable users to easily control and regulate appliances with the use of IoT-enabled sensors [3,4,5,6], therefore enhancing energy efficiency and reducing wastage.

In factory settings, especially in co-fabrication spaces, energy conservation by human activities is a crucial issue. As identified by the editorial of Volume 1 of this Special Issue [7], how advanced sensors can be integrated from the smart environment with human activity recognition and how one can be further connected to the energy consumed by these human activities provide a way to allow users to be aware of the consequence of their activities and further change these activities for the sake of energy conservation.

Co-fabrication spaces can monitor various building features, such as sensors, software, and online connectivity, analyze data, and generate information on usage patterns and trends that can be used to optimize the environment and building operations [8,9,10,11,12]. It is important to note that co-fabrication users have different needs compared to typical users, as they require dynamic and flexible spaces. Since co-fabrication spaces have a high level of user turnover, the common methods for evaluating usage and rent are not suitable. As a result, prototyping requires a specifically designed energy-saving strategy.

In co-fabrication spaces, rapid prototyping is critical during the user’s prototyping process, as it involves high energy consumption stages. During the prototyping process, the user typically works independently and attempts to solve practical problems or refine designs through a trial-and-error process. The emergence of rapid prototyping has lowered the fabrication threshold and increased the production of innovative designs, while also providing feedback on machined parts based on knowledge of the actual fabrication process [13,14].

Presently, many fabricators, designers, and researchers use digital devices, content, teaching guidelines, and even videos to learn, understand, and obtain information about prototyping to reduce the threshold for contact [15,16,17,18]. However, there are hidden technical operation issues and professional divisions of labor in the fabrication process that force designers to hand over design drawings to other professionals. This not only increases the design threshold and energy consumption but also causes the designer to spend more time consulting professionals and waiting for fabrication, while preventing the creation of different methods and allowing other experts to intervene in the design.

Collective data fusion approaches are critical in improving the efficiency and effectiveness of design processes that involve multiple disciplines. Such approaches can help in implementing experimental interactive installations and using innovative design methods. Traditional processes can be time-consuming and inefficient and require tailor-made construction processes for fabrication and assembly to test proposed prototypes. One example of a collective data fusion approach is the System of Design–Fabrication–Assembly (ViDA). This approach facilitates co-fabrication, cooperation, and information transmission. The ViDA system provides a platform for designers, engineers, and builders to share data and collaborate on projects, improving the efficiency of the design process. Another approach is the use of the Internet of Things (IoT) to collect data on building operations. Although this approach may not necessarily lead to behavior change, it can help to collect data on the building’s energy consumption, air quality, and other environmental factors. These data can be used to identify areas for improvement and optimize building operations. To improve the accuracy of sensing and help users manage the process between virtual data and physical environments, a proposed control mechanism called the human behavior sensor can be used. This mechanism uses gestures, behaviors, and interfaces to enhance the precision of the fabrication process and provide a smooth user experience. The use of a camera on another manipulator can allow for viewpoints to be executed at appropriate times during a task, ensuring that there is always a robust view suitable for monitoring the task. Collective data fusion can enable more efficient and effective design processes by incorporating these approaches. The integration of various design tools, technologies, and disciplines can enable designers to create innovative solutions that are more efficient and effective than traditional approaches.

### 1.2. Motivation and Approach

The purpose of this study is to integrate the Design–Fabrication–Assembly (DFA) architecture with the real-time interface and smart energy information in the digital twin. The motivation for the project is to improve the usage rate of the facilities of the co-fabrication space, to reduce unnecessary prototyping for users, and to maximize the use of dynamic energy for power consumption. The advantage of the DFA approach is that it can improve the fabrication and design process. DFA is mainly used for new innovative designs, and there is no practical example yet. Many applications in the materials or assembly are unknown, and design and manufacture need to take into account how the finished product will be assembled, and the process requires constant trial and error. The digital twin is a way to connect the virtual model and the physical, finished prototype. If the virtual and physical model can be integrated into a digital interface for designers to interact with, the problem of an digital communication medium in DFA can be solved, and the final prototype can be shown in advance in real time and can be used in a variety of materials.

Through further discussion, the development and problems in the three directions of human behaviors, digital system energy integration, and co-existing fabrication can be understood. The designer interacts with the transmission of digital information, energy consumption forecast, and physical fabrication through behavior, which can be embedded in the process of fabrication systems. Through the interaction of the above three directions and integrating the co-existing fabrication system for a smart DFA environment based on energy fusion information, a tool that supports designers, manufacturers, and assemblers in digital fabrication behaviors through virtual–physical integration technology can be created. In this project, it was hypothesized that every user could have an agent system, and the agent could change the effect of the device according to the user’s actions. Users do not have static identities and have multiple uses. Its behavior cannot be calculated using a single ID. Dynamic calculation and analysis are required. In this way, the user can understand not only her consumption and daily behavior, but also her investment in generated energy to improve the user experience. The tool system will provide construction method suggestions for users in the digital fabrication process to modify and manufacture in real time, and perform an interactive fabrication process of coexistence of virtual and real through the interactive interface combined with the real-time information of the system to solve (1) the designer’s concern about fabrication differences in understanding, (2) physical objects that can be augmented with virtual information, (3) problems with assemblers during component assembly, and (4) energy consumption alert and representation.

## 2. Related Research

### 2.1. Virtual Environment Energy with the Co-Fabrication Space

Smart environments connect computers and other smart devices to everyday settings and tasks, extending pervasive computing and promoting the idea of a world connected to sensors and computers [19]. These sensors and computers are integrated with everyday objects in people’s lives and are connected through networks. Human activity recognition (HAR) is the process of interpreting human motion using computer and machine vision technology. Human motion can be interpreted as activities, gestures, or behaviors, which are recorded by sensors. The movement data are then translated into action commands for computers to execute, and human activity recognition code is an important and effective tool [20,21,22]. The composition of co-fabrication spaces requires smart sensors and apps to create opportunities for more in-depth environmental monitoring compared to formal monitoring networks and to involve the public in environmental monitoring through participatory data collection and monitoring systems [23].

As electronic devices and their applications continue to grow [24], advances in artificial intelligence (AI) have also greatly improved the ability to extract deeply hidden information for accurate detection and interpretation. Additionally, current architectures must accommodate the number of devices dynamically available in the smart environment and the high degree of data heterogeneity to match the complexity of human activity. A key feature expected of backbones connecting smart environments is reliable communication that ensures lossless and low-latency data transmission over the network. Another aspect is to monitor the state of smart devices, handle error cases, and reallocate resources to maintain overall system performance [25,26].

Therefore, HAR proposed in this research contains three major technologies: (1) smart devices [24,26] for human interface and data collectors, (2) an agent-based system (an acting role model for its dynamic role model features [27,28]) as the software framework for communication and coordination among smart devices, and (3) Internet of Things (IoT) technology [29,30] for a hardware framework to collect signals from the physical environment and fabrication devices.

The Sensible Energy System (SENS) [30] configures an agent communication framework, which includes Human Agents, Devices, Local Servers, Consumption Managers, Communication Protocols, Location Servers, Power Switches, and Cloud Servers. Every user is a human agent, and a human agent uses a device in physical space. Once the local server receives the user’s information, it interacts with other agents in the system environment through its sensor module, execution module, and communication module. Building upon the existing work in the attribute-based access control model, it is possible to capture the physical context through sensed data (attributes) and perform dynamic reasoning over these attributes and context-driven policies using technology to execute access control decisions. By leveraging the existing structure, it becomes easier to access and capture user data.

**Figure 1:**
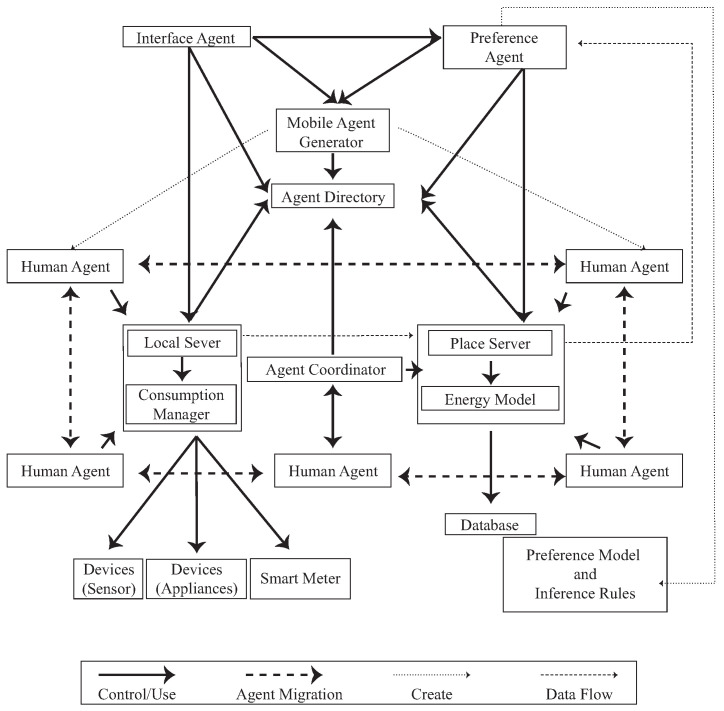
The concept of the user behavior dataflow in the SENS virtual environment [30].

Mixed reality (MR) not only occurs in the physical world or virtual world, but it also includes augmented reality technology that combines reality, and VR real-time interaction technology [8,31]. We can use an agent to describe event simulations in combination with physical objects placed in a virtual environment. Users in different roles can manipulate physical objects as physical counterparts to different machines and equipment in virtual space in real time. The system will visualize engineered factories, data, and behaviors and perform further analysis. This method combines dynamic discrete-event simulation seamlessly connected to physical objects placed in the virtual environment to enable co-design. The bridge between simulation and physical objects is made through a digital integration platform. Using physical artifacts as counterparts to the simulation’s virtual objects, participants can safely interact with the simulation regardless of their skill or ability. This design provides a framework by which many participants can engage in the process, experiment with the concept (Figure 1), and immediately respond to the adaptation of interactive surfaces.

### 2.2. Sensing the Physical Environment with the Smart Factory

The integration of technology and intelligent services is essential for most ubiquitous intelligent automation systems, especially those that require agile situational management, such as smart homes. Smart environments (SEs) evolve from ubiquitous computing following the idea that a physical world is richly and invisibly interwoven with sensors, actuators, displays, and computational elements, embedded seamlessly in the everyday objects of our lives, which are emotional and connected through a continuous network [19,32]. A smart environment provides a large amount of data because it consists of multiple heterogeneous sensors placed throughout the environment. A scalable and flexible platform integrates such devices and provides applications with the necessary interfaces to interact with information from available resources. Therefore, current architectures must accommodate the number of devices dynamically available on the smart environment and the high degree of data heterogeneity. A key feature expected of backbones connecting smart environments is reliable communication that ensures lossless and low-latency data transmission over the network. Another aspect is to monitor the state of smart devices, handle error cases, and reallocate resources to maintain overall system performance [25,26].

In the co-fabrication space, rapid prototyping is critical in the prototyping process of the user, and it is the stage that consumes the most energy. In the prototyping process, the user mainly works by himself and through practical problem-solving or design experimentation to find solutions. The emergence of rapid prototyping not only decreases the original fabrication threshold but also increases innovative design production. Presently, many manufacturers, designers, and researchers use digital devices, content, teaching guidelines, and even videos to learn, understand, and obtain information for prototyping to decrease the threshold of contact. However, there are some hidden technical operation issues and the professional division of work in the fabrication process that forces the designer to hand over the design drawings to other professionals to complete. When the fabrication process is divided, it not only increases the design threshold and energy consumption but also prevents the creation of different methods and allows other experts to intervene in the design.

**Figure 2:**
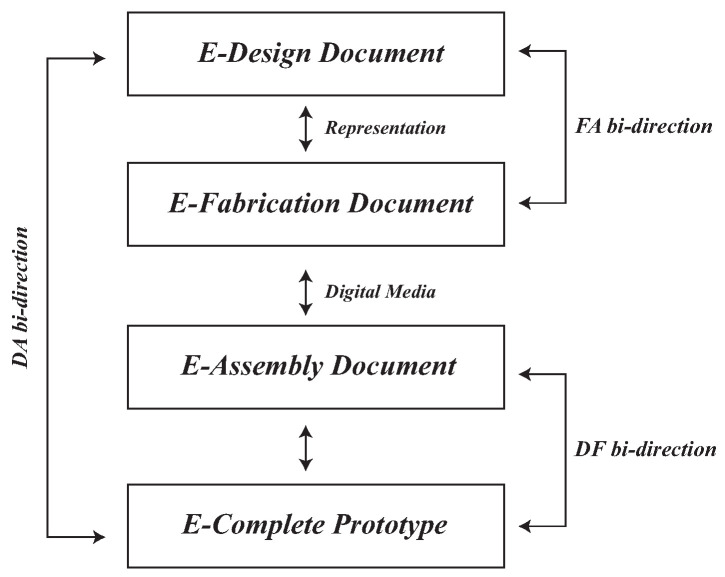
The Design–Fabrication–Assembly (DFA) process.

Every design is a unique and innovative structure that affects the prototyping process’s results. Therefore, the concept of customized small-batch production has become popular. Such development not only allows designers to create many different unique and special designs but also enables designers to undergo continuous trial and error and adjust processes, improving the realization of their ideas. If we can structure the trial–error process and anticipate possible problems in advance, it will reduce the number of attempts by designers to make mistakes. Among others, such a concept can be explored through the proposed DFA framework (Figure 2). DFA is a collective method that systematically maps out the prototyping process of an interactive interface in three stages: (1) the design stage, in which a device prototype is designed, (2) the fabrication stage, in which device components are tested and produced, and (3) the assembly stage, in which the components are assembled and tested [33]. To assist designers in reviewing designs and presenting more specific design concepts, 3D technology has further developed Augmented Reality (AR), Virtual Reality (VR), and Mixed Reality (MR). It is a simulation of 3D sketches on the computer, which allows designers to preview the real size, position, and angle of the virtual model. MR is the integration of both real and virtual worlds to generate new environments and visualizations in which physical and digital objects coexist and interact instantly [34,35,36].

### 2.3. Human Interaction and Behaviors with the Co-Fabrication Space

**Figure 3:**
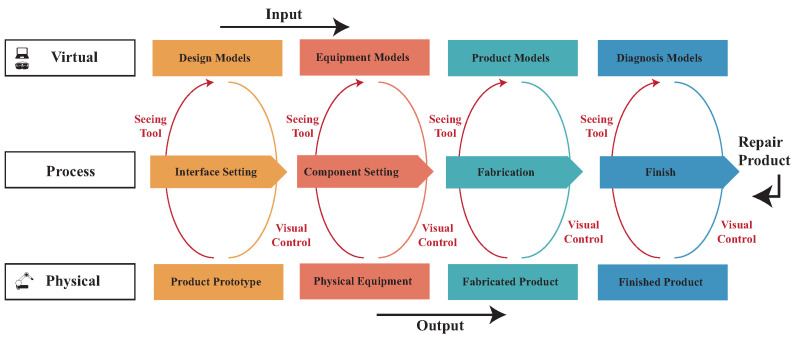
The concept of the visualization system of the DFA (ViDA) process.

The presence of human beings in various processes introduces a problem as they cause a source of disturbance and unpredictability in the form of performance variation. In the traditional digital fabrication process, designers ignore differences in the fabrication process due to unfamiliarity with the workmanship during the design-to-assembly process. In the process of making prototypes, the effect of different methods on different materials and the differences between various materials will affect the result of the actual work. Despite humans’ natural ability to be flexible, their presence still serves as a disturbance within the system. This makes modeling and optimization of these systems considerably more challenging and, in some cases, even impossible. The most common scenario is that the machine is unable to produce the design components, resulting in repeated errors. To enable many ideas in the larger intelligent manufacturing paradigm to be realized, it is crucial to overcome the significant challenge of improving the ability of robotic operators to adapt their behavior to variations in human task performance [37]. ViDA is part of the refinement in the co-fabrication space problem encountered and revolves around the DFA model [38]. The system suggests methods for the model, such as grouping components, wiring groups, and setting sensor positions. It generates several fabrication documents and assigns them to several different machines through different file types to let the user finish this document. Finally, when the fabrication documents are completed and enter the assembly stage, the assembler (Figure 3) comes into play. By the same token, the Co-Existing Fabrication System (CoFabs) [39] aims to enable the cyber-physical integrated system to extend the rapid prototyping application to other fabrication processes and show information on the MR device.

AR and MR are rapidly growing in popularity as mainstream technologies that can be used to develop intelligent settings that combine the physical world and virtual items, providing new options for supporting both solitary and group human activities. Augmented reality refers to a medium in which digital information is added or superimposed onto the real world in accordance with the world itself and shown to a user depending on their position and perspective [40]. In an interactive simulation, human behavior is used as the basis for interaction, rather than deliberate interaction, allowing users to further integrate into the experience and enhance the immersion of the interaction through a variety of different behaviors, as well as by performing system functions in a wearable manner [41,42,43,44,45].

To allow the maker to program and operate the fabrication tool more quickly and intuitively, a simple operation is accompanied by complex programming, forming the interaction between the human and the fabrication tool. The MR environment is a way to decrease the designer’s fabrication threshold, as the designer can immerse themselves in the MR environment, interact with virtual objects with direct gestures, and even introduce existing physical objects into their designs. The concept of ViDA [38,39] is currently a system operating on the common design software named Rhino [46]. The system can provide dynamic simulation, physical, structural, and environmental analysis, microcontroller programming, and fabrication tool programming, calculate and coordinate component numbers, component attributes, and binding numbers through imported geometric models, and split the prototype structure to generate corresponding mechanisms and component assembly maps. However, it can be hard to imagine the size and effect of the design on a computer screen. Thus, the ViDA system disassembles the design process and generates different stage-specific documents to help different users join the project. When making prototypes, common 3D modeling systems give designers operational control over prototype size and assembly. Fabrication documents, such as assembly sequence, component number, and sensor location maps, provide construction method suggestions in the prototyping process, improving the quality of the prototype. Such technology can make the self-created achieve an immersive effect.

## 3. Co-Existing Space for the Smart Factory

The aim of this project is to help users manage their activities and identify potential sources of energy consumption based on their choices, activities, and behaviors. Although there are many ways to track user activity and display energy information, they are limited in their effectiveness in changing user behavior and providing visualizations in a timely manner. The co-existing fabrication system for a smart DFA environment based on energy fusion information proposes new avenues for the design and interaction for a smart environment system that optimizes energy recommendations. With this in mind, the following design criteria are proposed for the development of the SENS+ system.

**Recognition of human behaviors**: The designer interacts with the transmission of digital information, energy consumption forecast, and physical fabrication through behavior, which can be embedded in the process of fabrication systems. Through the interaction of the above three directions and the integration of the co-existing fabrication system for a smart DFA environment based on energy fusion information, a tool that supports designers, manufacturers, and assemblers in digital fabrication behaviors through virtual–physical integration technology can be created.

**Recommendation of energy behaviors:** The project aims to improve the usage rate of the facilities in the co-fabrication space, reduce unnecessary prototyping for users, and maximize the use of dynamic energy for power consumption. The proposed DFA design framework aims to improve the fabrication and design process. DFA is mainly for new designs, and there is no practical example for many applications in materials or assembly, which requires constant trial and error. The digital twin is a way to connect the virtual model and the physical, finished prototype. If the virtual and physical models can be integrated into a digital interface for designers to interact with, the problem of the analog communication medium in DFA can be solved, and the final prototype can be shown in advance in real time and can be used in a variety of materials.

**User interaction and information visualization:** It is hypothesized that every user could have an agent system, and the agent could change the effect of the device according to the user’s actions. Users do not have static identities and have multiple uses. Their behavior cannot be calculated using a single ID and requires dynamic calculation and analysis. In this way, the user can not only understand their consumption and daily behavior, but also understand their investment in generated energy to improve the user experience. The tool system will provide construction method suggestions for users in the digital fabrication process to modify and manufacture in real time, and perform an interactive fabrication process of coexistence of virtual and physical through the interactive interface combined with real-time information.

### 3.1. The System

**Figure 4:**
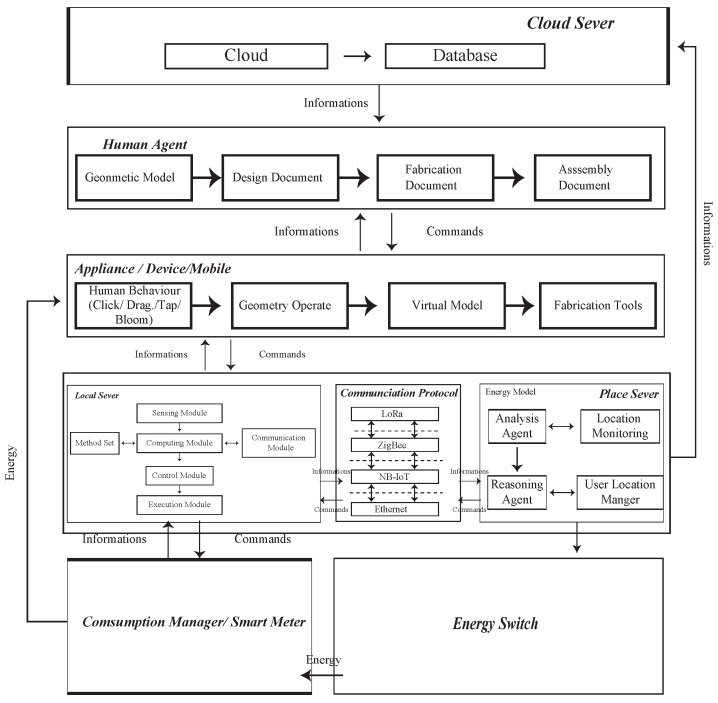
The system structure of this project.

Every design is a unique and innovative structure that affects the process of prototyping results. Therefore, the concept of customized small-batch production has become popular. This development not only allows designers to create many different, unique, and special designs but also enables them to undergo continuous trial-and-error and adjustment processes, improving the realization of their ideas. If we can structure the trial-and-error process and anticipate possible problems in advance, we can reduce the number of attempts made by designers to rectify mistakes. SENS+ provides a framework in which many participants can engage in the process, experiment with the concept, and respond to the adaptation of interactive surfaces. Figure 4 shows the SENS+ system structure, which is different from SENS [30] in terms of human agent and application design. In the SENS+ design process, the user will provide information and documents to the system, which will produce corresponding documents for different role users. SENS+ will focus on construction method suggestions, document readability, and user communication results. During the prototyping process, the user will undergo an iterative try–error process to improve their design. The backward modification of the design is a decentralized fabrication feature, so the prototyping process requires continuous communication to reach a consensus before completion [33]. Finally, SENS+ will (1) record the entire process and use the user behaviors and location data to give them future appointments for energy suggestions, (2) analyze the user-provided file and generate possible construction method suggestions for the user, (3) give the user machine suggestions when they choose a method, and (4) allow the user to set the machine function based on their requirements or suggest settings based on the system’s recommendations.

### 3.2. The Virtual Environment with the Agent and Co-Fabrication Communication Framework

The scenario awareness system is an important component of a smart environment, essential for conducting research. It involves obtaining various signals from the environment and users via home automation [47,48] to provide user services that meet their needs. The acquired situational information is converted into user needs through inference technology, and this process is called a situational awareness service. The activity perception system is an important research field that uses the external context to determine the user’s current internal activity context using perception and reasoning, including two main sub-studies of activity recognition and activity prediction. Automation can significantly improve the efficiency of penetration testing [48,49,50,51]. The proposed structure used IoT to present a penetration testing methodology, and its automation is based on the belief–desire–intention (BDI) model, which is one of the classical cognitive architectures of agents to evaluate IoT security. The BDI model provides a method to distinguish between choosing a plan from a library of options or an external planner application and carrying out already-active plans. As a result, BDI agents can balance the time spent thinking through ideas and deciding what to do with the time spent carrying out those plans. A third task, which is left to the system designer and programmer, is making the first blueprints, which are not covered by the model.

**Beliefs:** Beliefs represent the informational state of the agent, i.e., about the world, including itself and other agents. This stage includes inference rules and allows forward chaining to lead to new beliefs. Beliefs are literals of first-order logic, representing information about the world, updated by the perception of the environment and by the execution of intentions. Using the term belief rather than knowledge recognizes that what an agent believes may not necessarily be true and, in fact, may change in the future. Beliefs are stored in a database, sometimes called a belief base or a belief set, although that is an implementation decision.

**Desires:** Desires represent the motivational state of the agent and represent objectives or situations that the agent would like to accomplish or bring about. Desires can correspond to the task allocated to the agent and are usually considered logically consistent. Two kinds of desires are usually adopted: To achieve a desire, expressed as a belief formula, and to test a situation formula, which is a belief formula or a disjunction and/or a conjunction of them. Achieving goals involves practical reasoning, test goals involve epistemic reasoning. A goal is a desire that has been adopted for active pursuit by the agent. Usage of the term goals adds the further restriction that the set of active desires must be consistent.

**Intentions:** Intentions show the agent’s deliberate state, or what the agent has decided to do. Intentions are aspirations to which the agent has made a commitment. Plans are sets of instructions, recipes, or related knowledge that an agent can use to achieve one or more of its goals. Plans can merge with other plans, and they are often only partially formed at first, with details being added as the project moves forward. Each plan in the preset library of plans available to BDI agents contains multiple components. The trigger serves as a plan’s invocation condition by defining the event that the plan is meant to handle. If the agent detects an event in the event queue, it will consider the plan relevant. The context specifies, as a situation formula, when the plan is applicable, i.e., when the plan should be considered to form an intention. When a plan is filled out, goals are added to the event queue, after which other plans that can deal with similar events are considered. A plan may also include certain “maintenance conditions”, which specify the conditions that must endure for the plan to continue to be carried out. For both successful and unsuccessful implementations of the strategy, a set of internal actions is prescribed.

**Events:** Events serve as the agent’s reactionary activity triggers. An event could change goals, set off plans, or change beliefs. Externally produced events may be collected by sensors or other integrated systems. Additionally, internal events may be developed to activate disconnected updates or activity plans. Events in a queue are mapped to perception. There are three different types of events: receiving a message, acquiring a desire, and acquiring a belief. Events are implemented as structures that keep track of past data, such as the responses made to them and their success or failure. The transmission and reception of messages are used to put BDI agents’ MAS learning skills into practice.

**Figure 5:**
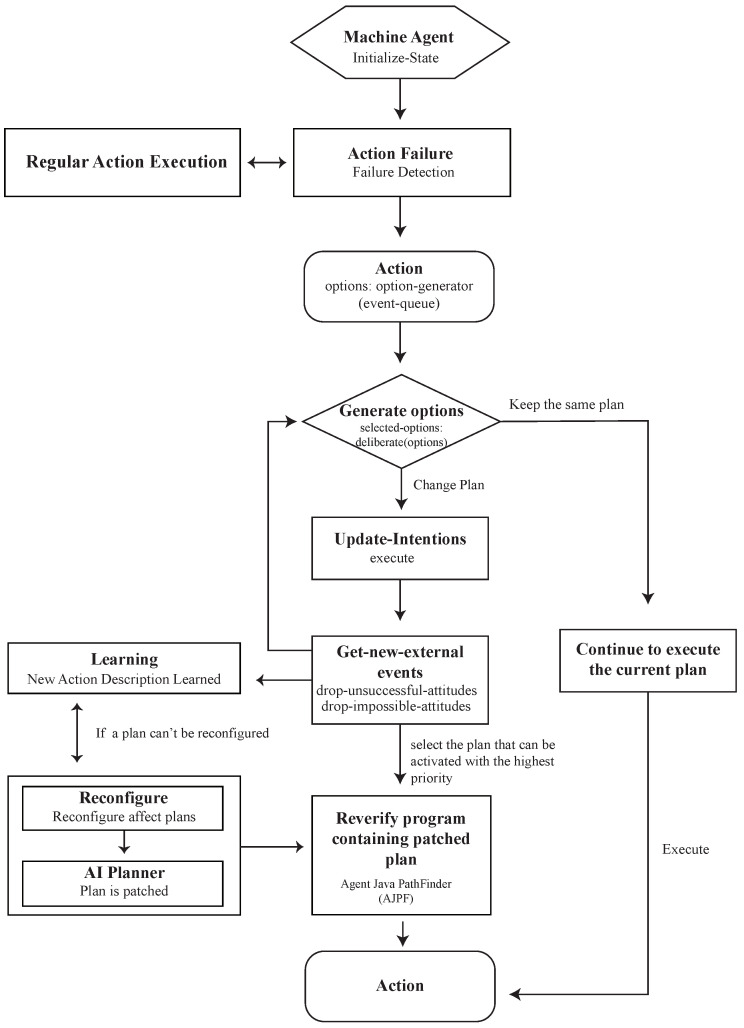
The concept of the communicate process in SENS+ smart sensor agents.

To integrate the originally separated virtual and physical spaces, some researchers [5,28,52,53] have used field observation, focus groups, and participatory observations to analyze user behaviors. The research used a series of methods to record and analyze their behaviors to design a suitable user interactive structure and usability evaluation. Simulating, predicting, and learning behavior can be done by adopting the Acting Role Model (ARM) [28], agent technologies (SENS) [30], intelligent dynamic interactions with the design system (DARIS), and BDI evaluation and learning [49,54,55,56,57,58] to examine interactive behaviors. The network entity system is generated by combining digital and physical information through the process of the embedded computer, network monitoring, and controlling the entity, calculating its feedback loop through the influence of the entity information [59], forming a co-existing interactive space. The researchers used smart sensors as sensor ports to create a coexistence space for sensory energies. In the environment, each physical object has its own environmental agent. The environmental agents in Figure 5 are divided into human agents, interface agents, preference agents, and sensitive agents. When a user enters the space, their local server starts tracking user behavior and device consumption. Consumption data and user behavior are sent to the Place Server and stored in a database for integration, calculation, and analysis. Finally, the information and details will be shown on the MR and App device.

### 3.3. The Physical Environment with a Smart Factory Architecture

**Figure 6:**
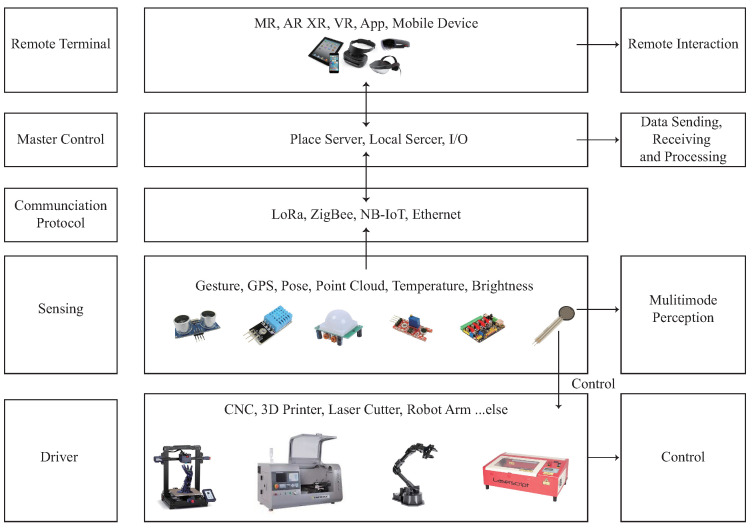
The concept of the architecture design of the SENS+ behaviorsystem.

There are many cases in which 3D modeling programs are used in engineering environments for the visualization and evaluation of design elements [60,61,62]. The information contained in the 3D modeling programs is necessary for presenting the design feasibility and for conducting research analysis. Rapid prototyping and 3D modeling programs allow for more direct and personal interactions with investors, designers, and other professionals involved in the project. The main function of rapid prototyping is to reduce the problems between the designer’s ability, the threshold, and operation time. Since the 1990s [63], many fabrication machines have required human control to carry out processes such as metal bending, sewing, and other fabrication tools. However, due to certain dangers in the fabrication process, professional skills are also required to operate these machines. Rapid prototyping aims to automate construction methods through prefab, with some degree of customization and support to avoid problems with usage. The convenience and rapid prototyping brought by digital fabrication have become the development trend of fabrication tools. However, for makers, the technical threshold of these machines is very high, and the manual assembly of parts is as complex as the parts constructed by fabrication tools [64,65]. In the early days, makers needed to communicate with manufacturers through drawings to confirm the physical finished product and subsequently hand it over to the manufacturer to produce the finished product, or even enter the factory to ensure the quality of the physical finished product. Therefore, it is necessary to consider a holistic approach to successfully incorporate fabrication tools, humans, and material mechanisms into the field. Digital fabrication focuses on single-task robots that can be deployed in the field, and designs a system to integrate the physical and virtual environment. The system is divided into a five-layer structure (Figure 6) including a remote terminal, a master control layer, a communication layer, a perception layer, and a driver layer, with the ultimate goal of designing construction sites that work similar to factories.

1. The remote terminal allows users to view information using a variety of tools, such as IoT Explorer, MR, XR, VR, App, and Mobile Devices, making it easy for them to understand the data.
2. The master control layer uses advanced technology such as the NXP RT1062, Raspberry Pi 3B+, and ibeacon to issue control commands and collect data from the entire system. The NXP RT1062 development board connects to Tencent IoT Explorer via an ESP8266 WiFi module to upload sensing data and receive remote control commands using the MQTT protocol stack. Additionally, the Raspberry Pi 3B+ development board’s network port communicates with radar to obtain point cloud data, which is then sent to the NXP RT1062 development board after processing.
3. The communication layer serves as the interface between the main control layer and the perception and driver layers. It provides several communication interfaces such as WiFi, LoRa, ZigBee, NB-IoT, Ethernet, and others.
4. The sensing layer contains three types of sensors: user gesture sensors, environmental state sensors, and energy sensors.
5. The driver layer receives commands from the main control layer and completes the movement control of the user, local machine, and device during the prototyping and design phase.

### 3.4. User Interaction in the MR Device

The fabrication process can pose a challenge for designers during the prototype fabrication phase. The lack of cleanliness in the fabrication process may lead to an inability to accurately evaluate the fabrication results of the prototype. To address this challenge, the Design–Manufacture–Assembly (DFA) design framework has been presented as an integrated prototyping process that allows users to leverage fabrication as part of a collaborative process. Schon and Wiggins [66] proposed a theoretical model called “Seeing–Moving–Seeing”, which is primarily used in design because it requires hands-on practice, creative thinking, design thinking, and observation. In this context, designers often allow their ideas to be converted and shown on the design media to better explain the circular thinking related to hands-on implementation and testing. However, as mentioned earlier, the fabrication process can sever the relationship between the designer and the finished product, making it difficult to fully apply the “Seeing–Moving–Seeing” model in digital fabrication.

**Figure 7:**
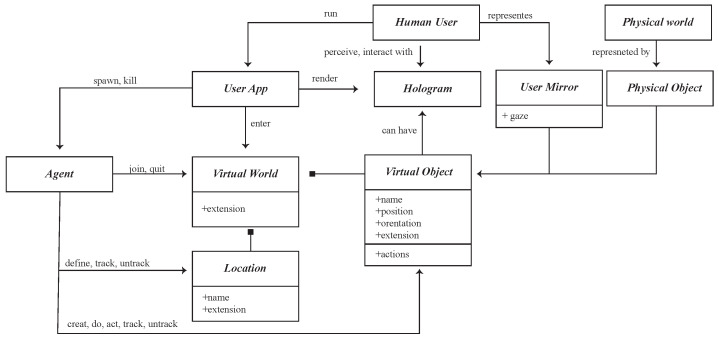
The concept of the co-existing human interaction process of SENS+.

In the DFA design framework, the designer first designs the virtual model through CAD/CAM, transfers the model to the fabrication machine for fabrication, and then takes the manufactured components back for assembly. During this process, the designer must keep trying and making mistakes to find the best solution. In this system, users may have varying levels of technical knowledge, including tool assembly and machine parameter adjustments. The information is displayed on both the user app and the HoloLens interface (Figure 7). Therefore, the system decomposes each step into different tasks and systematizes and modulates each step through design computing. Cyber-physical technologies are integrated into an interactive fabrication process that controls the physical machine to perform tasks, lowering the technological threshold. All hand icons are converted to gesture input, and all non-hand icons are automated. To perform an action, the user first selects the appropriate icon and then performs a gesture or waits for the system to complete its task. Virtual objects displayed in the user interface (UI) can have three different states. When the gesture icon is not selected, objects that cannot be modified are shown in light gray. When the object can be modified, the object turns yellow, and the object currently being modified is displayed in green. This color display provides information about the current system state. Feedback is provided, especially when grabbing objects, to help users determine whether the system recognizes their engaging gestures and how their movements affect the scene. Through these features, users can perform digital fabrication through simple gestures, integrating the real world with the virtual environment to create new environments and visualization effects. This allows reality and virtualization to coexist and interact immediately.

## 4. Evaluation of User Behavior in Co-Fabrication Space

The co-existing fabrication system for a smart DFA environment integrates real-world and virtual environments, as well as energy usage, based on the agent concept of a system. This system references physical entities and virtual information to each other in a recursive way through a series of physical changes, information analysis, generative fabrication, and energy design suggestions. This approach optimizes the workflow and can be applied to various types of users.

The purpose of the DFA design framework is to improve the fabrication and design process, which is mainly for new designs. However, there is no practical example of DFA applications in materials or assembly, and design and fabrication must consider how the finished product will be assembled, which requires constant trial and error. The agent is a way to connect the virtual model and the physical finished prototype. By integrating the virtual and physical models into a digital interface for designers to interact with, the problem of the analog communication medium in DFA can be solved, and the final prototype can be shown in real time and used in a variety of materials.

To integrate virtual and physical models, the system combines cameras and infrared sensors in a co-existing fabrication system to build an intelligent system of an intelligent DFA environment. The research uses the Seeing–Moving–Seeing–Design–Thinking model to help users refine their ideas. This process of the SENS+ system can be divided into two recursive sequences. One creates a virtual interface layer on top of the physical user perspective, constructing an environment with understandable information for the user. The other captures user gestures and movements to control a robotic arm for handcrafting or fabrication. Users can observe objects in physical space, interact with virtual objects, and recalibrate design properties during fabrication by comparing the appropriate hybrid environment with the virtual model.

To render the model in physical space and generate the model interface, Fologram, a Grasshopper plugin, transfers the geometric information to the SENS+ system and guides the machine end effector along the path. The research designed a UI and computer server system that generate fabrication tool code. The system communicates by scanning a QR code, enabling virtual models to generate data strings and return them to Grasshopper. Additionally, custom C# scripts perform fabrication calculations, such as path calculations that incorporate geometric data, behavioral variables, and remapping parameters between the fabrication tool and UI system. These processes allow for the efficient and accurate translation of virtual models into physical reality.

### 4.1. Human Behavior Data

The interaction between the user and the space connect devices can modify the user behaviors and change their usage and the consumption at the environment and co-fabrication configuration. A different user can use the devices to understand the operating procedures and energy usage. Table 1 represents an actual co-fabrication space scenario. In this co-fabrication space, the researcher observes and collects information regarding 10 users to analyze their weekly usage in a co-fabrication process in real time.

**Figure 8:**
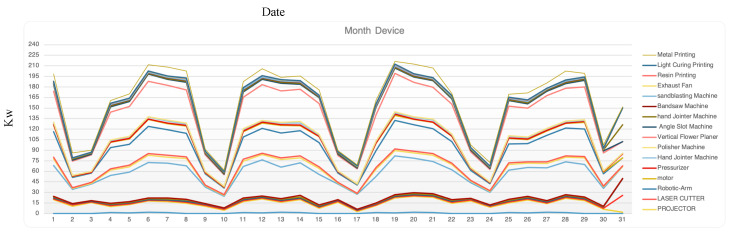
Monthly usage of every device.

The research models a variety of human activities using recorded data mining and machine learning approaches, sensor-based activity recognition in the environment, and the developing field of sensor networks. In Figure 8, the X-axis is the record date, the Y-axis is each device’s consumption energy in a month, and the lines of different colors represent different devices. The app on the user’s mobile phone connects to the environment sensor network for energy consumption predictions, and the environment records the user’s real position through the mobile phone’s GPS and beacon. Real-time tracking of the user’s entry and exit to the building, activity within the co-fabrication space, equipment usage patterns, and energy consumption is carried out. Researchers in the field of sensor-based activity detection believe that by equipping them with powerful computers and sensors that monitor the behavior of agents, these computers can better act on our behalf. HoloLens and other vision sensors that consider color and depth information enable more precise automatic action recognition and integrate a wide range of new applications in smart environments.

**Figure 9:**
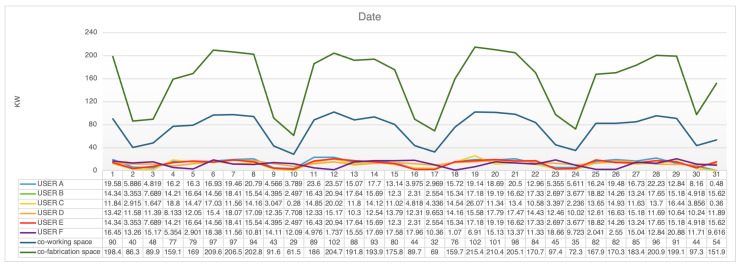
Different space compare data.

Figure 8 shows the recorded dates of each device in a month, while Figure 9 shows the daily usage of each user in the space over a month. The graphs indicate that usage still has a different effect, especially if a user stays in the same place each day. The system can use users’ demand to change their behaviors and day-to-day conditioning. Therefore, yearly records can help understand users’ introductory behaviors and usage. The value increases and decreases allow one to explore daily energy usage differences. In Figure 9, the green line represents co-fabrication space usage, while the blue line represents co-working space usage. It is easy to record the energy used by a single user in a space, but it is difficult to record and distribute energy in a co-fabrication space. This space includes more hidden user behavior, which is different from general usage. For example, one person may lease but divide energy consumption into subdivisions. To address this, the researchers identified users and their locations to help distribute energy usage and make personalized energy suggestions. Overall, the research shows that usage still has a different effect, especially if a user stays in the same place each day. By exploring daily energy usage differences, the system can help change user behaviors and encourage day-to-day conditioning. The yearly records can help understand users’ introductory behaviors and usage.

However, with the introduction of distributed storage, it becomes possible to balance the forecast and simulation of daily swings, such as the ones observed in the test research, with consumption patterns. The experiment has shown that human behaviors are complex and usage patterns are influenced by a variety of circumstances. Energy usage fluctuations throughout the day cannot be accurately predicted or specified. However, simulation feedback suggests that providing real-time access to consumption statistics may be a useful strategy for persuading users to alter their usage patterns. Ultimately, the results demonstrate that users can understand consumption data on their devices and that they need access to relevant information at all times.

### 4.2. User Interaction with the Scenario and the User Interface

**Figure 10:**
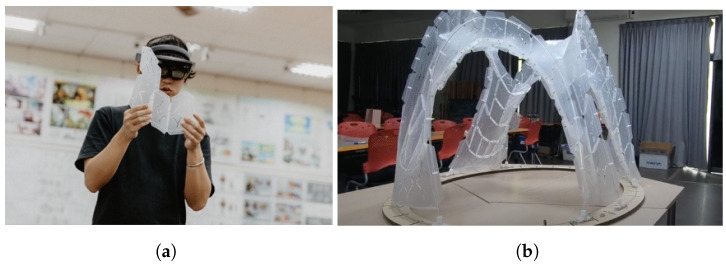
Users discussing the prototype and the final result. (**a**) Assemblers use HoloLens; (**b**) 300 × 300 × 300 prototype.

To gain insights into the practical implementation of the design, the researchers conducted an initial experiment using MR technology to superimpose and project complex shapes and shape grammar onto real-life environments. The experiment was divided into two stages. During the first stage (Figure 10a), user employed shape grammars to design 40 × 40 × 40 structures. As user designed and created small models, there were no issues with fabrication, assembly, or differences in construction methods or design comprehension, likely due to the models’ small size. In the second stage (Figure 10b), the user plans to scale up the design to a 300 × 300 × 300 funicular weaving structure with a folded plate skin design that has been calculated using shape grammar. However, the fabrication process may encounter some challenges due to the use of a single method, which can affect the assembly of individual components and make the construction process more complex. Consequently, designers will need to devote more time to describing the process and creating images to help others understand it. To address these challenges, users will evaluate and refine the fabrication and assembly methods to optimize the practical implementation of the design. This will involve considering multiple fabrication methods and potential solutions to potential assembly issues. By doing so, the user aims to ensure that the design can be successfully realized and applied in real-world scenarios.

**Figure 11:**
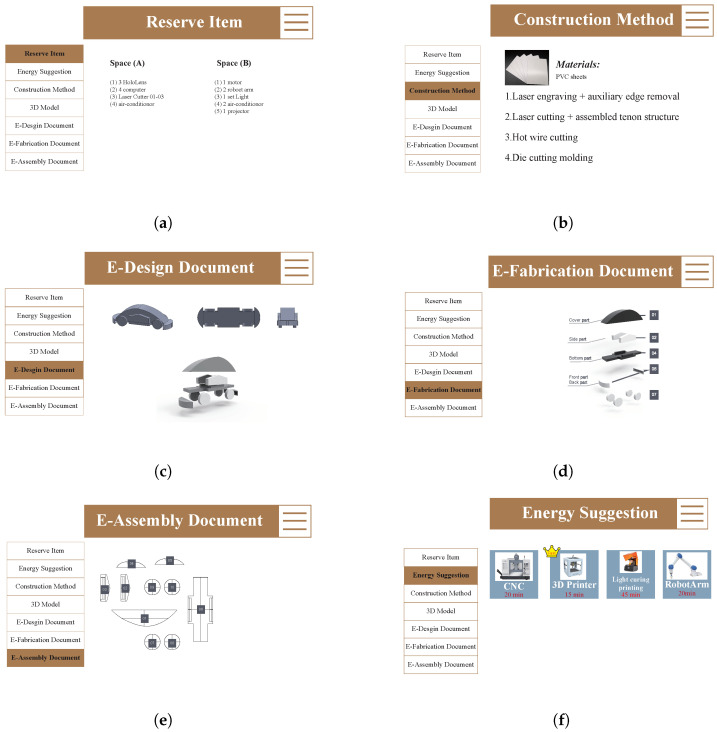
User scenario and UI. (**a**) Reserve Item; (**b**) Construction Method; (**c**) Design Document; (**d**) Fabrication Document; (**e**) Assembly Document; (**f**) Energy Suggestion.

Through the analysis of the actual operation process, the scenario is divided into two parts. The first part is the user app interaction, and the other scenario is the MR device. In the user app part, the user will use the agent to describe the interaction process. A modular individual reasoning architecture is represented by each agent. The benefits of that depiction are the simplicity with which operational reasoning or tactics for various interaction scenarios within the building may be put up, modified, and expanded. Users (Human Agents) must make a reservation on the machine they intend to use in a time slot with the appropriate dataset before they visit the area. In the co-fabrication scenario, a set of machine options must be selected, and the system must be given the power, speed, duration, and consumption for each machine. In the interaction scenario of SENS+ (Figure 11), users enter each space of the co-fabrication space with an app via the QR code, accessed via the screen on the door.

1. After scanning the QR code, the energy consumption, the generation of the location, and the reserve item are displayed in the system with basic location information (Figure 11a).
2. When the user approaches the electronic devices that are in the room, there are two ways to see the current consumption load of the device. First, through the system, it is displayed as energy consumption information on the user’s mobile device. Second, the demand and use are displayed on an MR with a notification sent to the user. Users can update their design to the system. The system will automatically analysis and disassemble the component and will provide a construction methods suggestion (Figure 11b).
3. After that, the system will generate three types of document for the user. First, an E-design document is generated by the designer 3D model. This document can help designers immediately examine their design (Figure 11c).
4. The e-design document is provided by the designer before fabrication, and this document can help the designer check their design directly. The second type of archive is the e-fabrication document (Figure 11d).
5. This file is provided for the fabricator to see. This stage is the file produced by solving the communication and imagination problems of the traditional digital fabrication process. These files will provide a detailed fabrication process and advice on how to break down each component. The last e-assembly document mainly provide the basis and step understanding for assembler assembly (Figure 11e).
6. Finally, the designer checks that their file is complete and can use their preferences and habits to choose their methods and fabrication machine (Figure 11f).

The energy suggestion is the system through which the 3D model file’s complex range provides the available machines and evaluates the consumption energy of different machines so that users can choose. When the machine completes the task, users can evaluate whether they are satisfied with the results by themselves. If the user is not satisfied with the results, MR will provide other options for construction method suggestions to the user. After the user evaluates and decides to switch to other construction method suggestions, they can make a new round of appointments through the app again. Through the app, users have the option to evaluate how much energy their chosen construction method can save, and the MR and app will tell users which devices are accessible in the area. In the scenario where the user evaluates the equipment usage and selects energy production, the system will display the energy usage as well as the estimated monthly savings from the energy source.

To create and test a behavior-based co-fabrication space environment, the system integrates smart sensing, a sensor network design, and user experiences. Within shared co-making spaces, the prototype integrates both energy generation and consumption. Each user’s unique usage patterns, habits, and energy use in the co-fabrication spaces have an impact on the everyday environment and energy usage. An option will also affect the consumption result because higher energy consumption occurs over longer periods of time, but the machine result will be more detailed, as desired by the maker. The optimization and standard options for the machine path recorded can provide energy consumption patterns as well as potential user awareness of the patterns. As a result, each agent will interact with one another, have a distinct mission and goal, and offer a chance for improvement to the user. The optimized alternatives consume less energy, even if they frequently do not produce optimal results.

## 5. Conclusions and Future Work

This paper presents an integration of the Co-Existing Interactive Fabrication Tool and proposes a Dynamic Interaction Process for Fabrication Design, a tool that supports designers, manufacturers, and assemblers in digital fabrication behaviors through virtual–physical integration technology. Linking the three phases of the DFA design framework and sharing information through the concept of digital twins allows the three phases to be continuously retraced and tested, enabling a real-time preview of the finished product in advance as well as a testing of multiple material applications. The tool framework illustrates the possibilities and flexibility of an immersive interactive environment and provides users with the ability to instantly view and confirm the production details based on the information provided. Through human–computer collaboration, the difference between the virtual and the physical in the cyber-physical coexistence space can be reduced, and the fabrication of complex spatial structures can be realized while reducing cost, risk, and complexity. The incorporation of method recommendations into the fabrication process can enhance user knowledge and ability for preparing fabrication. Users can discuss and adjust the graph and 3D model with fabricators immediately. The system provides a systematic perspective on disassembly, allowing fabricators to receive accurate fabrication documentation and complete accurate component fabrication in a multimodal environment.

In a future study, biosignal processing and activity modeling for multimodal human activity recognition [67] will provide further affective information needed for the SENS system. Additionally, the information-sharing function can also improve, such that other design members, participants from different fields, or remote designers can participate in the design or share that design together. The following three research directions are proposed.

1. **Fabrication process automation:** In the interactive fabrication process, fabricators are currently provided with the ability to design, manufacture, and assemble the operating components required for some of the fabrication processes one by one. After the fabrication is completed, the maker may then work out the path of the movement of the fabrication machine. To make digital fabrication easier to operate, design, and manufacture, in future development, the step-by-step design process can be automated through digital computing so that designers can control and prolong the design development process.
2. **Customized fabrication process integration:** In the current study, the fabrication process provided workflow recommendations that allowed individual machines to process and distribute, but it lacked a more precise workflow. On this basis, it is possible to extend the processing module through precise system calculations so that designers, fabricators, and assemblers can be involved in the same process by the integration of the tool, so as to achieve the aim of customizing the fabrication process integration.
3. **Digital twins for integrated method applications:** In the future, this study can focus on the fusion of real and virtual coexistence under the concept of digital twins. At present, spatial sensing as a mechanism feeds back physical products to the virtual environment, but only intercepts the current state for analysis, which is not in line with real-time physical signal transmission. The ability to receive signals in real time could be investigated in the future and combine sensors with interactive fabrication tools to allow physical feedback to occur in real time, making the finished product more compatible with virtual models.

## Figures and Tables

**Table 1:** Co-fabrication space usage.

Space	Machine	A	B	C	D	E	F	G	H	I	J
Laser Cutter Room	Laser Cutter	✓		✓				✓	✓		
	Prototyping	✓	✓	✓			✓	✓	✓		
	Cutting Machine		✓			✓	✓				
	Computer				✓						
	Laser Cutter Teaching				✓					✓	
3D Printer Room	3D Printer Class	✓									
	3D Printer	✓						✓	✓	✓	✓
	CNC Machine	✓						✓			
	Metal Printing							✓			
	Light Curing Printing							✓			
	Resin Printing										
Teaching Space	lecture		✓	✓		✓	✓	✓			
	Administration		✓	✓							
	Conference	✓	✓	✓		✓	✓	✓	✓	✓	
	Manager Meeting	✓	✓	✓		✓	✓	✓			
	Teaching Assistant Class	✓	✓	✓							
Muse Space	Project Discuss		✓	✓	✓	✓					
	Idea Thinking										
Scrub Room	Polisher Machine										
	Exhaust Fan				✓						
	Sandblasting Machine				✓		✓				
	Bandsaw Machine				✓					✓	
Metalworking Room	Hand Jointer Machine						✓				
	Angle Slot Machine		✓								
	Vertical Flower Planer		✓	✓	✓						
Model Room	Polisher Machine		✓								
	Hand Jointer Machine				✓					✓	
Robotic Room	Robotic Arm	✓			✓	✓					
	Motor	✓			✓	✓	✓	✓			
	Pressurizer	✓				✓	✓	✓			✓
